# Retroperitoneal pelvic tumours in women: diagnostic and therapeutic challenges

**Published:** 2020-03-27

**Authors:** GA Vilos, AG Vilos, J Hollett-Caines, B Abu-Rafea, GP Jacob, H Ettler

**Affiliations:** Department of Obstetrics and Gynecology, Western University, London, Ontario, Canada;; Department of Obstetrics and Gynecology, Chatham-Kent Health Alliance, Chatham, Ontario, Canada;; Department of Pathology, Western University, London, Ontario, Canada.

## Abstract

**Background:**

Gynaecologic pelvic tumours are very common and they can present with a variety of symptoms depending on their size, location, pathophysiology and histogenesis. Infrequently, some pelvic tumours are found in the retroperitoneal space presenting with similar symptoms. Our objective is to present our experience and review of pertinent literature on miscellaneous retroperitoneal tumours.

**Methods:**

Four women with retroperitoneal tumours (one schwannoma, one granulosa cell tumour and two hindgut (tail gut) cysts)) were encountered during routine laparoscopy (3 cases) and laparotomy (one case). Following multidisciplinary consultation and additional imaging, all tumours were removed by laparotomy with one case provoking litigation due to ureteral and bowel injury.

**Results:**

Using these four cases, and additional cases from the literature, we highlight the potential pitfalls and provide an algorithm to minimize risks and adverse clinical and legal outcomes associated with unexpected retroperitoneal tumours. The algorithm includes resisting the impulse/temptation to remove or biopsy these tumours, requesting intra-operative consultation(s), obtaining additional detailed imaging to characterize these tumours, providing appropriate counselling to patients, obtaining informed consent, and consulting the appropriate surgical teams. At times, an interdisciplinary approach may prove to be the best course of action in order to optimize treatment and ensure patient safety.

**Conclusion:**

If a retroperitoneal tumour is unexpectedly encountered, it is imperative to have intra-operative consultation (if available), to not attempt excision or biopsy, and to subsequently obtain post-operative multidisciplinary consultations, specific imaging, and information gathering in order to treat these heterogeneous masses as safely as possible.

## Introduction

Gynaecologic pelvic tumours are very common and can present with a variety of symptoms depending on their size, location, pathophysiology and histogenesis. Most of these are solid leiomyomas, followed by benign ovarian cysts. Infrequently, pelvic tumours can be found in the retroperitoneal space, separate from the uterus or the ovaries. Up to 30% of retroperitoneal tumours are asymptomatic and 50% will present with similar symptoms to intraperitoneal pelvic tumours; such as vague abdominal pain and distention, making distinction difficult (Alzaraa et al., 2008; Kurtz et al., 1986). During pelvic examination and/or imaging, retroperitoneal tumours can be cystic, solid or both. Retroperitoneal cystic lesions are rare, with an incidence of 1/5,750 to 1/250,000 (Guile et al., 2007).

Retroperitoneal solid lesions can arise from any of the mesenchymal elements including smooth muscle (leiomyomas), neuronal tissue (schwannomas), and others. We have previously reported a case and review of retroperitoneal leiomyomas and no further description will be provided here (Poliquin et al., 2008).

Since retroperitoneal tumours are so rare, general gynecologists invariably encounter them very infrequently during routine laparoscopy or laparotomy performed for leiomyomas or benign adnexal cysts. Faced with such unexpected tumours, gynecologists are consequently challenged on what the next most appropriate action is. The options at this time are to either attempt to excise the tumour, or at least take a biopsy for histologic assessment, or abandon any further surgical intervention and arrange for interdisciplinary consultation, including additional imaging and investigation.

Since the differential diagnosis of retroperitoneal tumours is fairly vast, the methods of treatment and their clinical implications can differ greatly depending on the pathology. Imaging modalities such as ultrasound, computed topography (CT) and magnetic resonance imaging (MRI) can be of benefit when attempting to narrow-down potential diagnoses. Characteristics such as lesion shape, size, location, and presence/absence of septa, calcifications or fat is information that can help the health care provider arrive at a diagnosis or at least help to rule out certain pathologies.

Herein, we present the management and clinical outcomes of four cases of miscellaneous pelvic tumours; one schwannoma, one granulosa cell tumour and two hindgut (tail gut) cysts, and briefly discuss the importance of pre- and intra-operative management of any retroperitoneal lesions, to minimize or avoid the risk of adverse or catastrophic events. It is imperative to have intra-operative consultation (if available) and subsequent post- operative multidisciplinary consultations, specific imaging, and information gathering in order to treat these heterogeneous masses as safely as possible.

## Materials and methods

Informed Consent:

Patients number 1, 2, and 4 have signed consent to publish their cases. Case number 4 is in the public domain (Santos v. Traff, 1999 ABQB 630), and ethics approval or patient consent is not required.

### Case 1: Schwannoma

A 59-year-old Caucasian woman presented with vague pelvic discomfort and was found to have a large pelvic mass, detected on routine physical examination by the family physician. Notably, the patient was menopausal as of age 48 and had been on hormone replacement therapy (HRT) since that time. Ultrasound imaging revealed an 8.6 x 7.6 x 8.3 cm solid pelvic mass, thought to be either of uterine or ovarian origin. MRI measured an 8.5 cm solid, heterogeneous mass behind the uterus, separated by a thin fat plane, and neither ovary was identified. Post-contrast imaging was suggestive of a pedunculated or subserosal leiomyoma. A biopsy and possible laparoscopy were recommended for definitive diagnosis of the mass. The patient was assessed by a gynaecologist who suggested an exploratory laparotomy, with possible total abdominal hysterectomy (TAH) and bilateral salpingo-oophorectomy (BSO).

At laparotomy four weeks later, the mass appeared to arise from the sacrum and the rectosigmoid colon was noted to be adherent to the mass. The uterus, fallopian tubes, ovaries, and cul-de-sac were all normal. Intraoperative consults included a gynaecologic oncologist, who did not feel that the mass was of gynaecologic origin, and an orthopaedic surgeon, who proposed that the mass was most likely a chondroma and that further imaging should be obtained, prior to definitive surgical excision. At this time, no further surgery was performed and no biopsy was taken, given that the vascularity of the tumour was unknown.

Computed tomography (CT) scan of the lumbar spine described the soft tissue mass as extending to the anterior cortex of the S1 vertebral body and to the anterior margin of the L5-S1 disc. There was direct continuity of the mass with the S1 vertebral body, but no erosive or destructive changes. CT of the abdomen and pelvis noted again the 9 cm soft tissue mass, separate from the bladder and uterus. The possibility of a sarcoma was questioned. A subsequent MRI of the lumbar spine described the pelvic mass as intimately opposed to the upper sacrum, but not involving the vertebral bodies.

**Figure 1:**
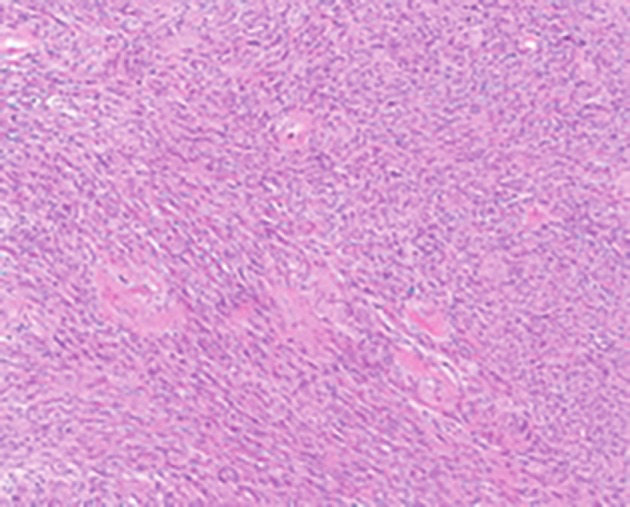
Schwannoma: Fasicles of spindle cells and hyalinized vessels (H&E, x100).

Two months after the first laparotomy, the patient had a second laparotomy, performed by a general and a vascular surgeon. The tumour was found to be adherent to the sigmoid colon, left ureter, left internal iliac vein (which was partially resected) and presacral fascia and veins. Intraoperative blood loss was approximately three litres and the patient was transfused with six units of packed red blood cells. The final pathology revealed a Schwannoma measuring 9.0 x 10.5 x 9.0 cm and weighing 364g (Figure 1). Post-operatively, the patient developed transient symptoms of sciatica but recovery was otherwise uneventful.

### Case 2: Granulosa Cell Tumour

A 49-year-old woman, P2G2, BMI 37 kg/m2, was referred to our clinic with chronic pelvic pain and a complex right adnexal mass measuring 8.2 cm, which had been followed for 4 years. She had a remote abdominal left salpingo-oophorectomy for ovarian torsion. On pelvic examination, a soft mass filled the posterior cul-de-sac which could not be dislodged from the pelvis. The CA125 was normal. The patient was scheduled for laparoscopic evaluation and possible right salpingo-oophorectomy and hysterectomy.

**Figure 2:**
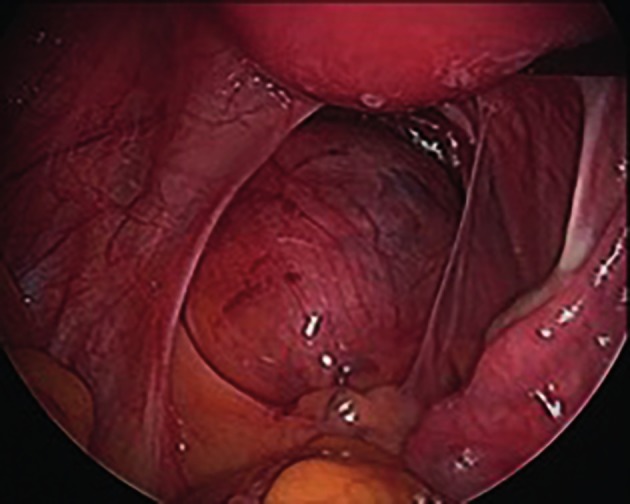
Retroperitoneal granulosa cell tumour.

At laparoscopy 3 weeks later, a normal sized uterus was displaced anteriorly by a retroperitoneal soft mass, filling the cul-de-sac, and intimately close to the sacrum, possibly arising from the rectosigmoid with significant blood vessels running over the mass. The right ovary and tube were normal and there was an absence of the left adnexa (Figure 2). After intra- operative consultation with a general surgeon, it was decided not to attempt any surgical intervention at the time, including no biopsy, and the plan was to re-evaluate this tumour with additional imaging and biopsy under CT guidance. Endometrial curettage was performed and the endometrium was reported as disordered proliferative endometrium.

One month later, a CT scan reported a 9.0 x 6.9 x 6.9 cm complex cystic mass interposed between the anterior rectal wall and posterior margin of the uterus. This lesion was thought to be unusual for a tailgut cyst, since it was not centred in the retrorectal space. Six weeks later, an MRI measured the mass as 7.4 x 5.2 x 8.4 cm, relatively well circumscribed, cystic, and with some solid components. A US guided biopsy was reported as neoplastic tissue, possible sex cord stromal tumour.

**Figure 3:**
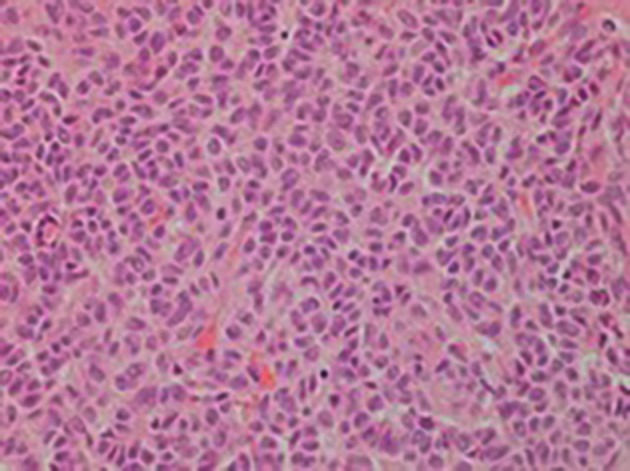
Granulosa cells with typical nuclear grooves (coffee beans. H & E X 200).

At laparotomy four weeks later, the tumour was relatively easily dissected from the anterior wall of the rectum, posterior wall of the vagina and the left pelvic side wall. The final pathology was reported as granulosa cell tumour, adult type with lesional cells present at the resection margins (Figure 3). After review at the tumour board, it was decided that no further treatment was indicated but to follow this patient with annual MRI for at least 10 years.

Six months later, the patient was seen with abnormal uterine bleeding (AUB). Endometrial biopsy indicated proliferative endometrium and the patient was treated with a LNG-IUS. At 3 years of follow-up, she remains amenorrheic and well with no evidence of recurrent disease.

### Case 3: Hindgut or Tail gut cyst

A 45-year-old Caucasian woman had a CT scan for vague pelvic discomfort, which detected a right presacral, hyperdense, smooth-appearing lesion measuring 4.8 x 3.8 cm. The possibility of a neurogenic tumour was raised. MRI described the mass as a right presacral, complex cyst with high signal intensity measuring 5.9 x 3.9 cm, and appeared to be adjacent to the S1 foramen ovale, but separate from the nerve root. Ultrasound demonstrated a right posterior, complex mass measuring 5.5 cm, which was suspicious for a dermoid cyst.

**Figure 4:**
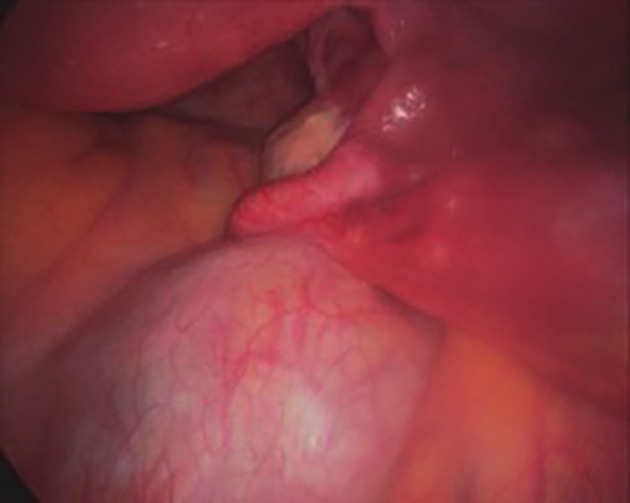
Retroperitoneal Tail gut (hindgut) cyst.

**Figure 5:**
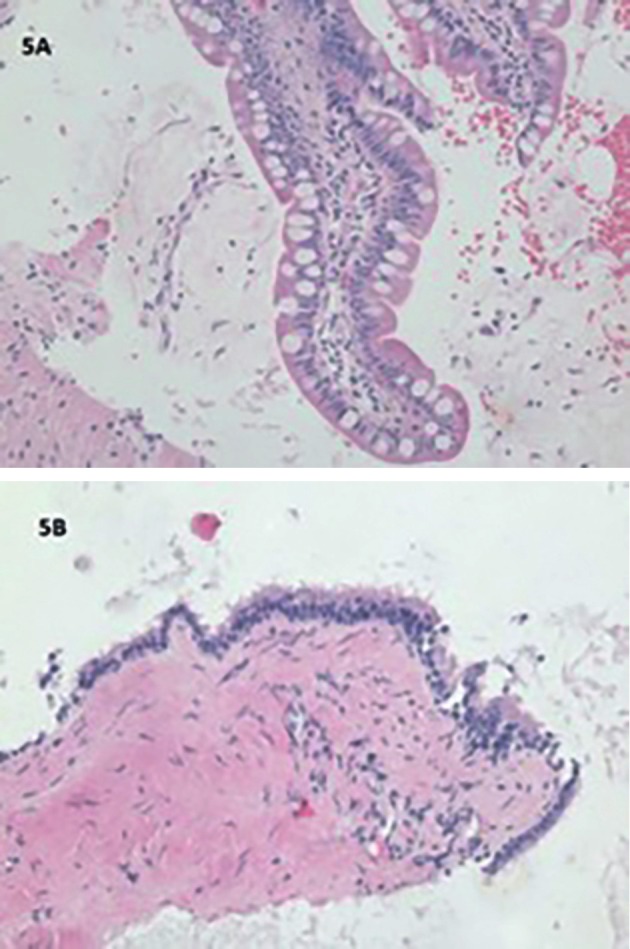
Tail gut cyst biopsy showing intestinal mucosa with Goblet cells in the left (H&E, x200) and respiratory epithelium in the right picture (H&E, x200).

At laparoscopy three weeks later, the mass was clearly retroperitoneal and separate from the pelvic organs (Figure 4). No biopsy was taken. After appropriate multidisciplinary consultations and imaging, a CT guided biopsy of the mass was obtained three months later, which demonstrated small intestinal mucosa (Goblet cells) and fragments of smooth muscle (Figure 5A). Thinking that this was an inadvertent bowel biopsy, a second biopsy was done two months later which indicated necrotic tissue with small intestinal mucosa and smooth muscle (Figure 5A, H&E, 200x), and ciliated, respiratory-type epithelium (Figure 5B, H&E, x200).

As the patient was experiencing neurogenic-type pain, and there was a possibility of malignancy, the retroperitoneal cyst was excised by laparotomy 6 weeks later by a general surgeon who had specialized in pelvic surgery. At the time of surgery, the mass was noted to be soft, somewhat fluctuant, and fixed to the right pelvic sidewall. The mass was related to the right sympathetic nerve bundle and autonomic plexus, but did not involve major vascular structures.

Microscopically, the cyst was lined by ciliated, respiratory-type epithelium with a smooth muscle wall with areas of focal squamous metaplasia. The diagnosis was a benign epithelial cyst, consistent with duplication cyst or hindgut cyst. Additional details of this case have been published previously (Vilos et al., 2013) .

### Case 4: Hindgut cyst

This is a medicolegal case in the public domain (Santos, 1999).

A 49-year-old, morbidly obese woman presented with left lower quadrant pain and a cystic mass identified on ultrasound measuring 7 x 8 x 13 cm. Past surgeries included a total abdominal hysterectomy and a subsequent bilateral salpingo- oophorectomy.

At a laparotomy three weeks later, it was unexpectedly found that the mass was a retroperitoneal cyst. The gynecologist decided to dissect the cyst by creating a plane between the capsule of the mass, even though the ureters were not visualized. Dense adhesions made the dissection difficult and there was limited visualization of the operative field due to the patient’s large body habitus. Eventually, the mass was removed and a drain was left in the pelvis.

On post-operative day #2, the smell of urine was detected in the drain and ureteric injury was confirmed with retrograde pyelogram. The patient subsequently underwent a laparotomy and repair of the left ureter. However, she continued to have symptoms and, due to fecal material present in the abdominal drain, a rectosigmoid injury was suspected. On post-operative day #7, she had a colostomy for a uretero-rectal fistula. The fistula healed spontaneously and the colostomy was reversed one year later. The final pathology was hindgut cyst.

Expert opinion was that the gynecologist should have sought assistance from a urologist intraoperatively to help with identification of the ureters. The legal outcome was negligence on the part of the gynecologist for damage of the ureter, but not for damage of the bowel.

## Discussion

### 

As stated in the introduction, almost all these retroperitoneal tumours are encountered during routine laparoscopy or laparotomy to remove common pelvic tumours such as leiomyomas or adnexal cysts. Under these circumstances, gynecologists invariably are faced with several challenges including whether or not to excise an undiagnosed tumour without patient informed consent, or to at least take a biopsy of the mass, or to abandon any further surgery and re-operate at a later date after further evaluation and discussions with the patient and other health care professionals.

### Biopsy/Excision of solid lesions

*Schwannoma:* The present case of schwannoma, and review of similar published cases, indicate that a biopsy of solid tumours can result in excessive bleeding, disseminating/upstaging disease if the lesion is malignant and, occasionally, removing a perfectly normal ectopic organ such as a pelvic kidney. In our case, the schwannoma was found to be adhered to the left internal iliac vein which required ligation and resection and resulted in blood loss of at least 3 litres.

Schwannomas are tumours that arise from the neural crest-derived Schwann cell. They are associated with neurofibromatosis type 2 and are mostly benign, with only 1% becoming malignant. Schwannomas are usually well-circumscribed, encapsulated masses that are usually attached to the nerve and symptoms are caused by local compression of the involved nerve (Cotran et al., 1999). Depending on the location of the lesion, surgical resection is the most common treatment. Safe laparoscopic removal of schwannomas in 3 women with intractable vulvodynia and coccygodynia has been reported (Possover et al., 2013).

Under these circumstances, excision or biopsy may not be appropriate, and additional tests and imaging should be performed to determine the nature of the lesion. An image-guided biopsy can always be performed at a later date after further interdisciplinary consultations, detailed discussions with the patient, and complete informed consent have been completed.

*Granulosa Cell Tumours:* In our case, we have no explanation for the origin of this granulosa cell tumour. In a previously reported case and review of the literature of granulosa cell tumors found on the uterosacral ligaments, we included the different theories on the histogenesis of ectopic granulosa cells (Pun et al., 2012).

The first possibility is that development of extraovarian, sex cord–stromal tumors and supernumerary ovaries are limited to structures that differentiate close to the mesonephros or along the path of ovarian descent into the pelvis (Griffith and Carcangiu, 1991; Smith et al., 2000). Primary granulosa cell tumors and supernumerary ovaries have been found on the adrenal glands, retroperitoneum, broad ligament, and pelvic sidewall (Cruikshank and Van Drie, 1982). None of these have been found deep in the pelvis in the rectovaginal space.

A second possibility is that the granulosa cells arose from a focus of endometriosis or endosalpingiosis. Uterine tumors resembling ovarian sex cord tumors are a well-described entity in which endometrial stromal cells show sex cord–like differentiation at histologic analysis (Czernobilsky, 1982). Typically, they manifest as a large uterine mass and are mistaken for a leiomyoma. One study reported on a focus of sex cord tumor, with annular tubules, in association with endometriosis in the fallopian tube (Griffith and Carcangiu, 1991). No concurrent ovarian disease was noted. Thus, the possibility of sex cord differentiation from ectopic endometrial stromal cells (endometriosis) was hypothesized. However, as in our case, no disease was noted in the uterus and the residual adnexa. Findings at preoperative ultrasound, laparoscopy, and endometrial biopsy were all within normal limits.

A third possibility is that these ectopic granulosa cells may have been released from a normal ovary, with subsequent implantation in a dependent area of the pelvis such as the cul-de-sac (Paul et al., 2009; Naniwadekar and Patil, 2010; Sakai, 2007).

Generally, granulosa cell tumors of the ovary have a favorable prognosis following treatment taking into account the patient’s age, needs and the stage of the disease. Complete laparotomic or laparoscopic excision of the tumour without rupture and in-bag morcellation, including hysterectomy and bilateral salpingo-oophorectomy, together with staging (peritoneal washings, biopsies, and infracolic omentectomy) is the mainstay of treatment. Unilateral salpingo-oophorectomy coupled with staging and endometrial sampling to exclude metastatic disease and/or concurrent endometrial pathology can be offered to women of childbearing age with early-stage (stage Ia) disease. There is no consensus on whether radical surgery should be performed when these patients have completed their childbearing or when they reach menopause. Adjuvant chemotherapy is currently recommended only in those cases with advanced, recurrent or metastatic disease. Long-term surveillance is mandatory including clinical follow-up and tumor markers (i.e. Anti-Müllerian Hormone and inhibin B), as disease may recur many years after the initial diagnosis and treatment.

### Biopsy/excision of retroperitoneal cystic lesions

Cysts that develop in the retrorectal space are histologically heterogeneous, as this space contains multiple embryological remnants as well as neuroectoderm, notochord, and hindgut (Bullard Dunn, 2010). For the majority of retroperitoneal cysts, the treatment of choice is complete surgical excision (Lu and Tseng, 2010). However, as demonstrated in our case 2 & 3, a more cautious course of action is occasionally required to minimize or avoid potential complications.

*Hindgut Cysts:* Tailgut and duplication cysts are types of retrorectal tumours. Their true incidence is unknown, but in one study, they were found to arise in approximately 1/40,000 hospital admissions (Jao et al., 1985). Tailgut cysts are usually multiloculated and are thought to arise from a portion of the embryonic tail that fails to regress. However, duplication cysts are unilocular and lined by epithelium similar to that of the gastrointestinal and respiratory tracts (Prasad et al., 2000). The vast majority of these retrorectal tumours are benign and most require surgical management (Bullard Dunn and Rothenberger, 2008).

Puncturing, biopsy or attempted excision of an uncharacterized pelvic cystic lesion, however, may also result in catastrophic events if it is vascular, if it is related to an expansion of the dural sac, or if it is related to the spinal cord. Therefore, following determination of a cystic lesion in the pelvis by imaging (CT, US, MR), it might be wiser to plan for complete surgical excision. Although we have not encountered any neurogenic cysts in our practice, we summarize two cases reported from the literature to highlight their importance.

Anterior Sacral Meningocele: An anterior meningocele is a herniation of the dural sac through a defect in the sacrum. The sac is in continuity with the subdural space and, therefore, contains cerebrospinal fluid.

Berstock et al. (2008) described their experience with an 18-year-old woman with a 2-year history of pelvic pain, which was worse at the time of menses and associated with urinary frequency. A pelvic ultrasound described both ovaries and an additional cystic lesion measuring 8.3 x 8.4 x 9.0 cm, thought to be related to the right adnexa. At laparoscopy, a large retroperitoneal mass was noted and biopsy, rather than removal, was considered to be appropriate. However, an intraoperative general surgical consultation advised abandoning the procedure and obtaining additional imaging. An MRI revealed a large mass in the pelvis confluent with the dural sac, consistent with an anterior sacral meningocele.

Following referral to a spinal surgeon, the patient was counseled with respect to the risks of bowel, bladder, and sexual dysfunction, numbness, and intraoperative coning of the brain stem before surgery. Ligation of the meningocele sac was performed at the level of the communicating stalk, with the patient in a gentle Trendelenburg position, and 1L of cerebrospinal fluid was aspirated from the cyst. Postoperatively, the patient had some minor transient neurological symptoms which subsided within one year. Additional anterior sacral meningoceles, previously misdiagnosed as ovarian cysts, have also been reported (Tarlov, 1938; Benes et al., 2008; Vamsi et al., 2010).

Central to this case is an emphasis on advocating a cautious approach, when confronted with an incidental unknown pelvic retroperitoneal cyst, as deleterious consequences, including death from coning of the brain stem, may occur from puncture or biopsy of such a lesion (Berstock et al., 2008).

Tarlov Cysts: These cysts, also known as perineural cysts, were first described by Tarlov, in 1938, and are meningeal cysts that are filled with cerebrospinal fluid (CSF) (Tarlov, 1938). Tarlov cysts can be found anywhere along the spinal cord (cervical, thoracic lumbar) but are most frequent in the S1- to-S5 region, arising from the sacral nerve root near the dorsal root ganglion. They occur in 1% of the population, but are mostly asymptomatic. Again, in our practice, we have not encountered such a perineural cyst but a brief description of a case described by Hirst et al (2009) warrants attention to highlight the catastrophic events which can be associated with misdiagnosis and mismanagement of such lesions.

A 72-year old woman presented with a short history of poor urinary flow. Pelvic ultrasound noted a left-sided cystic lesion, measuring 4.2 x 3.9 x 5.4 cm, with echogenic material and fluid of questionable ovarian origin. Tumour markers were normal. The patient was subsequently booked for a laparoscopic bilateral salpingo-oophorectomy and cystoscopy.

At laparoscopy, the pelvic organs appeared normal, but a retroperitoneal cystic structure was noted on the left side, between the rectosigmoid and the lateral pelvic wall. The peritoneum was then divided, and the cyst punctured. The cyst drained clear fluid and a tiny biopsy was taken.

Immediately post-operatively, the patient complained of excruciating pain in the left hip and buttock with radiation down to the back of the thigh and calf. The patient also complained of burning, stabbing, and electric shock sensations in the same region without relief with major analgesics. An MRI showed multiple cysts within the sacrum and a complex 5 cm cyst with multiple septation and evidence of haemorrhage in the region of the sacral plexus. Histopathology of the laparoscopic biopsy from the cyst indicated neural tissue elements consistent with a Tarlov cyst.

The patient ultimately had a cerebrospinal fluid arachnoid drain placed at L2,3 to relieve the pressure and a laminectomy at L4,5 to remove clear fluid under high pressure. The nerve sheath root was narrowed with bipolar radiofrequency. The patient had good pain relief following these procedures, unfortunately, the voiding problems persisted (Tarlov, 1938).

Tarlov cysts are fluid-filled sacral perineural cysts arising at the junction of the dorsal root ganglion and the nerve root. They display the characteristic of delayed filling on myelography (not meningoceles). Tarlov cysts contain nerve fibres and ganglion cells in the cyst wall and develop between the endoneurium and perineurium of the nerve root. They typically require the expertise of a neurosurgeon and pain specialist.

The final point to highlight from these cases is that attempts to dissect and remove these tumours without appropriate preoperative evaluation, patient counselling and consent, planning, and multidisciplinary intra-and post-operative consultations can result in significant adverse events or sequelae, which may provoke litigation with unfavourable legal outcomes. This is evident in case No.4 (hindgut cyst) where a difficult dissection of the cyst resulted in ureteral and rectosigmoid injury, while in case No. 1 (schwannoma), although the dissection was more difficult, the use of appropriate planning and consultation resulted in uneventful clinical outcome.

Limitations of this study include that it was conducted in a single center with a limited number of patients.

## Conclusion

**Figure 6:**
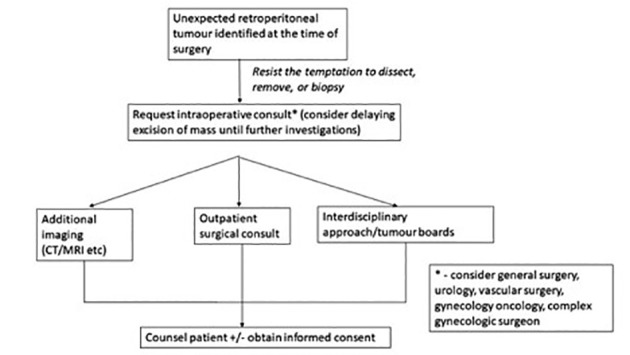
Algorithm for the approach to an intraoperative unexpected retroperitoneal tumor.

Retroperitoneal tumours, while an uncommon entity, can pose both diagnostic and therapeutic challenges. With these four cases, and additional cases from the literature, we highlight the potential pitfalls and provide an approach to minimize risks and adverse clinical and legal outcomes associated with unexpected retroperitoneal tumours. Our recommendations include: resisting the impulse/ temptation to remove or biopsy these tumours, requesting intra-operative consultation(s), obtaining additional detailed imaging to characterize these tumours, providing appropriate counselling to patients, obtaining informed consent, and consulting appropriate surgical teams for definitive treatment as outlined in Figure 6. At times, an interdisciplinary approach may prove to be the best course of action in order to optimize treatment and ensure patient safety.
